# Cellulose Nanocrystals Reinforced Zein/Catechin/β-Cyclodextrin Inclusion Complex Nanoparticles Nanocomposite Film for Active Food Packaging

**DOI:** 10.3390/polym13162759

**Published:** 2021-08-17

**Authors:** Longwei Jiang, Yanlong Han, Xiangyi Meng, Yawen Xiao, Huajiang Zhang

**Affiliations:** 1 College of Engineering, Northeast Agricultural University, Harbin 150030, China; hanyl@neau.edu.cn (Y.H.); menxy97@126.com (X.M.); yawenxyw@163.com (Y.X.); 2 College of Food Science, Northeast Agricultural University, Harbin 150030, China

**Keywords:** cellulose nanocrystals, zein, antioxidant activity, biodegradable films, food oxidation

## Abstract

In this study, following the green, environmentally friendly and sustainable development strategy, cellulose nanocrystals (CNCs) were prepared through a solvent-free esterification reaction between microcrystalline cellulose and maleic anhydride, combined with subsequent ultrasonic treatment, and maleic-anhydride-modified CNC-reinforced zein/catechin/β-cyclodextrin inclusion complex nanoparticles nanocomposite films were prepared by a facile solution casting. The amount of CNCs in the film matrix was 0–8 wt%, and their effect on structural, physicochemical and functional properties of the resulting films were investigated. SEM images showed that the addition of CNCs made the microstructure of the film more smooth and uniform. The intermolecular hydrogen bonds between CNCs and film matrix were supported by FT-IR. XRD analysis also confirmed the appearance of a crystalline peak due to the existence of CNCs inside the films. The incorporation of CNCs significantly reduced water vapor permeability, water solubility and the swelling degree of the nanocomposite film, and also significantly increased tensile strength and elongation at break from 12.66 to 37.82 MPa and 4.5% to 5.2% (*p* < 0.05). Moreover, nanocomposite film packaging with CNCs can effectively inhibit the oxidation of soybean oil.

## 1. Introduction

Nowadays, food packaging plays an important role in food processing and the food supply chain, which must meet the increasingly stringent requirements and needs of society [1]. Most of the materials used in the food packaging industry are synthetic polymers obtained from petrochemical products, which cause serious environmental problems due to their non-degradability [2]. Therefore, the development of environmentally friendly polymers with higher biodegradability has become a general trend.

Zein, which displays biodegradability, thermoplasticity and excellent film-forming properties, has been extensively investigated as a commercial material for edible packaging [3]. However, like many other proteins, the brittle film formed by natural zein is not flexible enough to withstand industrial processing [4], and the tensile strength of single zein-based film needs to be improved [5]. In recent years, researchers have made great efforts to improve the physical and chemical properties of zein films [6,7,8]. Fortunately, the application of nanotechnology has provided new horizons for the development of biopolymer-based food packaging materials. Biopolymer-based nanocomposite films can be used as carriers for functional additives, such as antioxidants and antibacterial agents, and the added nano-reinforcing phase can improve the mechanical and barrier properties of packaging materials [9]. Catechin (CA) is a kind of plant polyphenol extracted from tea and other natural plants and has important functions of antioxidant and free radical scavenging activities [10]. Cyclodextrins (CDs) are non-toxic macrocyclic oligosaccharides, consisting of (α-1,4)-linked α-D-glucopyranose units, with a hollow hydrophobic interior and hydrophilic outer surface [11]. This feature enables CDs to host both polar and non-polar guests of polymers or small molecules [12]. The most common CDs are α-CD, β-CD and γ-CD with 6, 7 and 8 glucopyranose units [13]. Many studies have reported that the antioxidant stability of CA was improved when combined with β-CD [10,12,14]. Our group previously prepared CA/β-CD inclusion complex nanoparticles (NPs) by the nanoprecipitation method [15]. The NPs do not need to be separated and dried, and the suspension can be used as a solvent for dissolving zein directly, which makes the process environmentally friendly and low cost. Compared with the single zein film, the elongation at break and tensile strength of the nanocomposite film increased from 1.52 to 4.55% and 2.28 to 12.49 MPa, respectively. After storage, the antioxidant activity of the nanocomposite film still maintained a high level. However, the performance of zein/CA/β-CD inclusion complex NPs nanocomposite film still has great potential for improvement.

Cellulose is considered to be a fascinating biopolymer, the subject of extensive development and research and a renewable raw material. Cellulose is composed of D-glucopyranose units, which are linked together by β-(1→4)-glycosidic bonds to form linear macromolecules with high crystallinity [16]. Cellulose nanocrystals (CNCs) are rigid rod-like particles with a typical acicular structure measuring 100–1000 nm in length and 4–25 nm in diameter [17]. CNCs combine the excellent properties of cellulose with the fascinating characteristics of the nano-sized materials [18]. As an eco-friendly, sustainable, green substance, CNCs also offer a potential for the reinforcement of biopolymer-based food packaging, e.g., protein and polysaccharide films, to replace petroleum-based materials [19,20]. CNCs have the advantages of non-toxicity, high crystallinity, and a large aspect ratio and surface area, which can effectively improve the poor water barrier and tensile strength of biopolymer-based films, and meet the requirements of different foods [21,22]. Yadav et al. (2020) recently developed CNC-reinforced chitosan-based sustainable biocomposite films, and they found the CNCs improved the water vapor permeability, mechanical and UV barrier properties of the biocomposite films [23]. Huq et al. (2012) prepared CNC-reinforced alginate-based nanocomposite film, and they found the incorporation of 5 wt% CNCs in alginate enhanced the tensile strength and water vapor permeability of the nanocomposite film [20]. Ma et al. (2017) studied chitosan film reinforced with modified CNCs as cellulose spheres for food packaging applications, and the composite film exhibited improved thermal and mechanical properties [24]. Chemical and mechanical treatments were commonly used for removing amorphous cellulose and obtaining CNCs [21,25]. During their processing, CNCs usually introduce some groups to negative charge, such as carboxylate and sulfate groups, which makes it easy to separate from raw materials and form stable dispersion due to electric repulsion [22]. According to the principle of electrostatic interaction, whether the matrix was charged or not, the negative charge of CNCs will have a profound impact on the performance of biopolymer-based films [26]. When CNCs with negative charge were added to the matrix, electrostatic repulsion was beneficial to the homogeneous distribution, even morphology and further contribution to film strength [27].

In this study, a simple, green and efficient method was used to prepare CNCs by the solvent-free esterification of microcrystalline cellulose with maleic anhydride combined with subsequent ultrasonic treatment. Then, various amounts of maleic-anhydride-modified CNCs were introduced into zein/CA/β-CD inclusion complex NP films by a facile solvent casting approach. The aim of this study was to investigate the effects of CNCs, as eco-benign renewable reinforcements, on the structural, physicochemical and antioxidant properties of nanocomposite films. Moreover, the effect of nanocomposite films on the oxidative of soybean oil was studied.

## 2. Materials and Methods

### 2.1. Materials

β-CD (diameter of 1.53 nm) was obtained from Sinopharm Chemical Reagent Co., Ltd. (Shanghai, China). Zein (average molecular weight of 25,000–45,000) was purchased from Dulai Biotechnology Co., Ltd. (Nanjing, China). Microcrystalline cellulose and maleic anhydride were bought from Aladdin Chemical Co., Ltd. (Shanghai, China). 1,1-diphenyl-2-picrylhydrazyl (DPPH) was purchased from Shanghai yuanye Bio-Technology Co., Ltd. (Shanghai, China). (+)-CA (purity > 98%) and 2,2′-Azino-bis (3-ethylbenzothiazoline-6-sulfonic acid) diammonium salt (ABTS) were purchased from Macklin Biochemical Co., Ltd. (Shanghai, China). Soybean oil was bought from Jiusan Grain and Oil Industry Group Co., Ltd. (Harbin, China). All other reagents were of analytical grade and purchased from Aladdin Chemical Co., Ltd. (Shanghai, China).

### 2.2. Preparation of Nanocomposite Films

The CNCs were prepared by the method described in our previous research with slight modification [28]. Briefly, maleic anhydride (15 g) and microcrystalline cellulose (3 g) were mixed in a mortar and then reacted at 120 °C for 3.5 h. The esterification reaction did not need solvents, which has the advantages of easy purification and low cost. After that, the reactants were washed with absolute ethanol and then purified water until the filtrate became neutral. The pH of the esterified microcrystalline cellulose suspension was adjusted to 11 using an aqueous solution of sodium hydroxide (1 M), and then washed with purified water until the pH reached 7.8. The purified water was added to the suspension until 0.5 wt% esterified microcrystalline cellulose suspension was obtained. The suspension (30 g) was ultrasonically treated with an ultrasound generator (SONICS VCX750, SONICS & MATERIALS Inc., Newtown, CT, USA) with output power of 600 W, and the treatment time was 30 min. The CNC suspension was centrifuged and freeze dried, and the obtained CNC powder was stored at 4 °C for further use. The crystallinity of the prepared CNC was 82.86%.

The zein film was prepared by referring to our previous research [15]. First, 1 mL of absolute ethanol containing CA (5.8 mg) was dropwise added into 10 mL of β-CD aqueous solution (2 mM), and the system was continuously stirred (200 rpm, 5 h) in the dark. Then, the nanoprecipitation method was used to prepare the CA/β-CD inclusion complex NPs after inclusion reaction. Absolute ethanol (44 mL) was added dropwise into the CA/β-CD inclusion complex solution under stirring. After that, the mixture was continuously stirred (200 rpm, 20 min) to form a NP suspension. Then, 1.6 g of zein was added to a 20 mL NP suspension and stirred (400 rpm) for 30 min. The solution was mixed with different contents of CNCs (0, 2, 4, 6 and 8 wt%) and glycerin (30 wt%) on a zein basis. Finally, the film solution (20 mL) was casted on a polypropylene mold (8.1 cm × 11.2 cm). All films were equilibrated in a desiccator (75% RH) for 2 days at room temperature. The nanocomposite films containing 0–8 wt% of CNCs were named Zein/NPs, Zein/NPs/2CNCs, Zein/NPs/4CNCs, Zein/NPs/6CNCs and Zein/NPs/8CNCs.

### 2.3. Structural Characterization of Films

Scanning electron microscopy (Zeiss, MERLIN Compact, Jena, Germany) was used to observe the surface morphology of the film. The film samples were fixed on the sample stages with conductive pastes, and then plated in a vacuum gold plating machine for 2 min. After that, the micro-structures of the films were observed under scanning electron microscopy (SEM). A Fourier transform infrared (FT-IR) spectrometer (IRAffinity-1 SHIMADZU, Kyoto, Japan) was used to record the FT-IR spectra of the film in the range of 4000–500 cm^−1^ with 32 scans at a resolution of 4 cm^−^^1^. Before testing, the sample was dried at 40 °C for 24 h, then the sample (1–2 mg) was ground with KBr (200 mg) and then compressed into an ultrathin disc for measurement. An X-ray diffractometer (Rigaku D/max2500, Rigaku Corporation, Tokyo, Japan) with Cu Kα radiation (λ = 1.542 Å) at 40 kV and 40 mA was used to record the X-ray diffraction (XRD) pattern of the film. The film samples were scanned between 2θ = 5° and 35° with a rate of 2°/min.

### 2.4. Determination of the Physical Properties of Films

#### 2.4.1. Thickness, Moisture Content (MC), Water Solubility (WS) and Swelling Degree (SD)

A helical micrometer was used to measure the thickness of the film (Harbin Measuring & Cutting Tool Group Co., Ltd., Harbin, China). The film was dried at 105 °C until constant weight and the mass loss of the film relative to its initial mass was the MC. The proportion of the film dry matter dissolved in water after immersion for 24 h was the WS. The SD was tested based on a previous method [29]. The film (40 mm × 10 mm) was immersed in distilled water (30 mL) for 24 h and the ratio of the weight of the film after absorbing water to its initial weight was the SD of the film.

#### 2.4.2. Water Vapor Permeability (WVP)

The WVP of the film was tested by the previous method [15]. The film sample was sealed over a special aluminum cup (1.3 cm in depth and exposed area of 28.26 cm^2^) containing 2 g of anhydrous CaCl_2_, and placed into a desiccator with RH of 95% at room temperature for 48 h. The WVP of the film was calculated using the following equation:

(1) $$\mathrm{WVP}\;\left(gm^{-1}h^{-1}{\;\mathrm{Pa}}^{-1}\right)=\frac{W\times t}{M\times D\times \Delta P}$$

where *W* is the weight change of the film (g); *t* is the thickness of the film (m); *M* is the lapsed time for the weight gain of film (h); *D* is the area of film (m^2^); Δ*P* is the change in pressure (Pa).

#### 2.4.3. Optical Property

A UV–Vis spectrophotometer (UV-2550, Shimadzu, Japan) was used to measure the light transmittance of a film sample between 300 and 800 nm and the absorbance at 600 nm. The films were cut into rectangle pieces (1 cm × 4 cm) and placed in a spectrophotometer cell. The air was selected as a reference. The opacity of the films was calculated using the following equation [23]:

(2) $$\mathrm{Opacity}={Abs}_{600}/d$$

where *Abs*_600_ is the value of absorbance at 600 nm and *d* is the thickness of the film (mm).

#### 2.4.4. Mechanical Properties

The dumbbell-shaped specimens with 4 mm neck width and 50 mm long were cut from the film samples. Mechanical properties, including elongation at break (EAB) and tensile strength (TS), were measured by a tensile testing machine (Model QJ 210, Shanghai, Qingji, China), based on our previous research [15]. The 100 N sensor was selected and the tensile speed was set to 10 mm/min.

### 2.5. Antioxidant Properties

The film samples were stored in a dark box (50 cm × 50 cm × 130 cm) at room temperature. At designated time intervals (3 and 90 days), films were taken out and the antioxidant activity was determined by the following method.

The free radical scavenging activity of the film was tested by DPPH assay [30]. The sample (1 cm × 1 cm) was put into test tube and immersed into a 0.2 mM DPPH ethanol solution (2 mL) for 30 min in the dark. The film was then separated from the solution by filtration, and the absorbance of the solution was measured at 517 nm. The DPPH radical scavenging activity of the film was calculated using the following equation:

(3) $$\mathrm{DPPH}\;\mathrm{radical}\;\mathrm{scavenging}\left(\%\right)=\frac{A_{0}{-A}_{i}}{A_{0}}\times 100$$

where *A_i_* and *A*_0_ are the absorbance of the sample and blank group, respectively.

The total antioxidant activity of the film was tested by ABTS assay [30]. The film (1 cm × 1 cm) was placed in diluted ABTS+ solution (2 mL, 6 min). The film was separated from the solution by filtration. Then, the absorbance of the solution at 732 nm was measured using a UV–Vis spectrophotometer (UV-2550, Shimadzu, Japan). The total antioxidant activity of the film was calculated using the following equation [15]:

(4) $$\mathrm{Total}\;\mathrm{antioxidant}\;\mathrm{activity}\;\left(\%\right)=\frac{A-A_{i}}{A}\times 100$$

where *A_i_* and *A* are the absorbance of the sample and blank group, respectively.

### 2.6. Oxidative Stability of Soybean Oil in Film Pouches

The effect of films on inhibiting oil oxidation was tested based on a previous method with slight modifications [31]. The film pouch (8 cm × 8 cm) containing 22 mL of soybean oil was heat sealed and stored at room temperature for 30 days. Every 5 days, 2 mL of oil was taken out and thiobarbituric acid reactive substances (TBARS) and peroxide value (PV) were analyzed. For PV, 5 mL of methanol/chloroform (1:2, *v*/*v*) was mixed with an oil sample (50 μL), and then reacted with of 20 mM ferrous chloride (50 μL) and 50 μL of ammonium thiocyanate (30%, *w*/*v*) in 3.5% HCl (*w*/*v*) for 20 min. The absorbance of the reaction solution was determined at 500 nm, and PV was calculated by using the cumene hydroperoxide standard curve. For TBARS, 5 mL of solution containing trichloroacetic acid (15%, *w*/*v*), thiobarbituric acid (3.75%, *w*/*v*) and 0.25 mM HCl was mixed with oil (1 g). The mixture was heated in boiling water (10 min), cooled and centrifuged (5000× *g*, 20 min). The absorbance of the supernatant was determined at 532 nm, and a 1,1,3,3-tetramethoxypropane standard curve was used to calculate the TBARS value.

### 2.7. Statistical Analysis

All tests were repeated at least three times and the results were expressed as mean ± standard deviation. Data were analyzed by the Duncan test with SPSS software, and *p* < 0.05 was statistically significant.

## 3. Results and Discussion

### 3.1. Structural Characterization of Films

#### 3.1.1. Morphology

**Figure 1:**
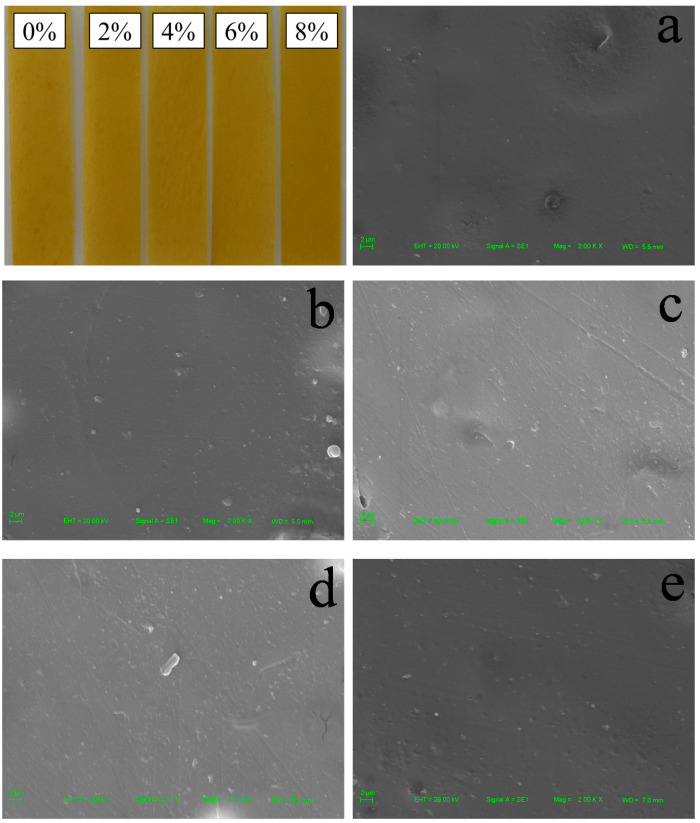
Physical appearances and SEM images of Zein/NPs (**a**), Zein/NPs/2CNCs (**b**), Zein/NPs/4CNCs (**c**), Zein/NPs/6CNCs (**d**) and Zein/NPs/8CNCs (**e**).

In order to investigate the effect of CNCs on the surface structure of zein/CA/β-CD inclusion complex NP film, the physical appearance and microstructure of the film were characterized. As shown in Figure 1, all zein films presented a yellow color. In addition, the macroscopical surface of zein films was smooth. With the increase in the amount of CNCs added, there was almost no visible agglomeration on the surface of the films. The micrographs are included in Figure 1. There were a few agglomerations on the surface of Zein/NPs without adding CNCs. The lines on the film surface were caused by scratches in the mold. The microstructure of zein films became smooth and homogeneous with the increase in CNC content, indicating that the zein and CNCs have good compatibility. There was no obvious agglomeration on the microscopic surface of the films, indicating that the esterified CNCs can be well dispersed in the matrix due to electrostatic repulsion, and this is conducive to the improvement of film strength [22]. Huq et al. (2012) also found a similar phenomenon that alginate film was more compact in the presence of CNCs, which improved the mechanical properties of the films [20]. Yadav and Chiu (2019) reported that the smoothness of the microstructure of κ-carrageenan film increased upon the addition of CNCs [32]. Yadav et al. (2020) also found that chitosan film containing CNCs showed a uniform and dense structure due to the small size and homogeneous distribution of CNCs in film, and the uniform and homogeneous distribution of CNCs in the nanocomposite film was the main reason for the improvement of physical and mechanical properties of chitosan-based film [23]

#### 3.1.2. FT-IR Spectra

**Figure 2:**
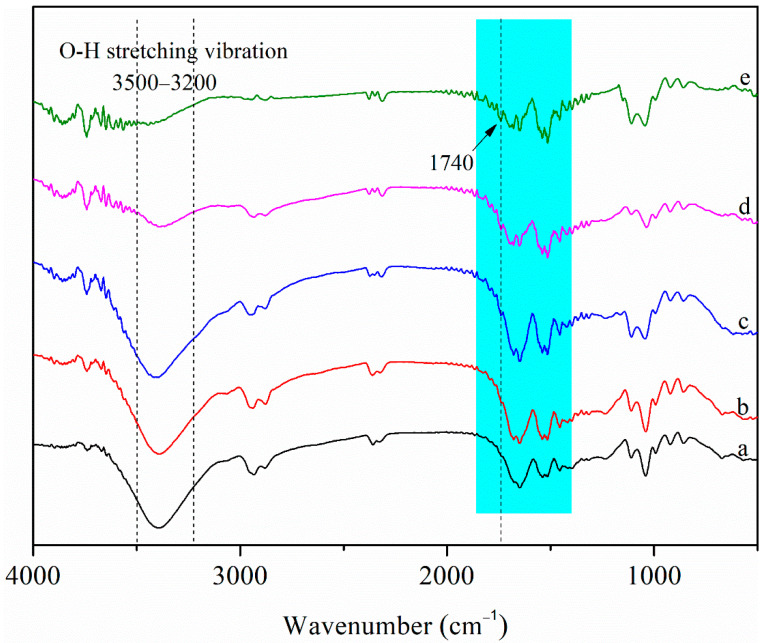
FT-IR spectra of Zein/NPs (**a**), Zein/NPs/2CNCs (**b**), Zein/NPs/4CNCs (**c**), Zein/NPs/6CNCs (**d**) and Zein/NPs/8CNCs (**e**).

Figure 2 shows the FT-IR spectra of the films. For zein, the amide II region between 1500 and 1600 cm^−^^1^ reflects C–N stretching vibration and N–H bending vibration and the amide I region between 1600 and 1700 cm^−^^1^ was generally characteristic of the C=O stretching vibration of peptide bond [33]. Generally, the amide II band indicates the environment for hydrogen bonding, whereas the amide I band represents secondary structures such as β-sheet and α-helix of the protein [34]. The intensity of the infrared spectrum of the nanocomposite films in the amide II band peak (around 1540 cm^−1^) increased after adding CNCs, indicating that the interaction between amide and CNCs changed the hydrogen environment. There was a wide peak around 3400 cm^−1^ in the amide A region between 3200–3500 cm^−1^, which corresponded to the O–H stretching vibration [6]. After adding CNCs to Zein/NPs, the absorption peak of the nanocomposite films between 3200 and 3500 cm^−1^ shifted. The result implied the potential interaction of hydrogen bonding among zein, zein/CA/β-CD inclusion complex NPs and CNCs. In addition, the infrared spectrum of the nanocomposite films at 1400–1800 cm^−1^ displayed many hetero peaks with the increase in the CNC content. This was because the infrared spectrum of CNCs has characteristic peaks in the range of 1400–1800 cm^−1^ [28]; with the increase in CNC content, the characteristic peaks of CNCs gradually appear in the infrared spectra of the films. Notably, compared with the spectrum of Zein/NPs, a new peak at about 1740 cm^−^^1^ attributable to C=O stretching of the maleate moiety in esterified CNCs was observed in Zein/NPs/CNCs films [28], and the peak intensity increased with the increase in CNC content, indicating that CNCs were successfully embedded into the film matrix.

#### 3.1.3. XRD Patterns

**Figure 3:**
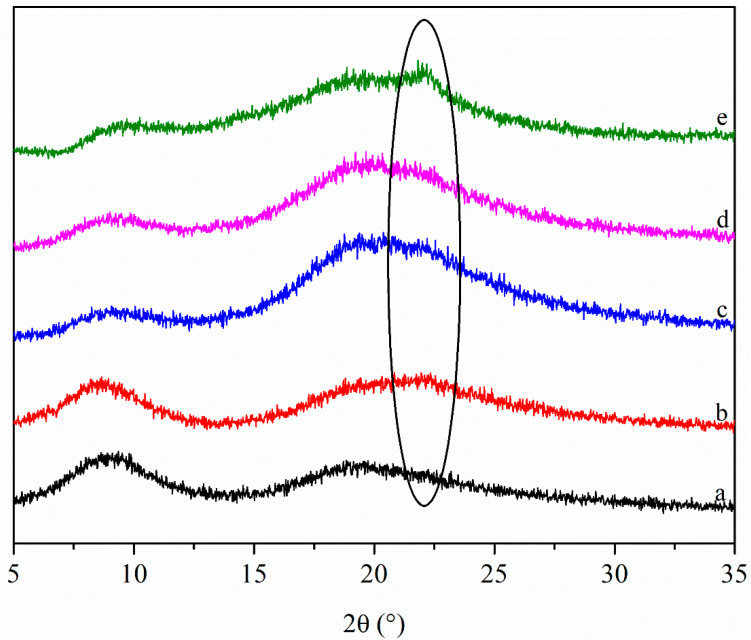
XRD patterns of Zein/NPs (**a**), Zein/NPs/2CNCs (**b**), Zein/NPs/4CNCs (**c**), Zein/NPs/6CNCs (**d**) and Zein/NPs/8CNCs (**e**).

The XRD patterns of the films are shown in Figure 3. There are two diffraction peaks in the XRD pattern of Zein/NPs, the peak positions are 9.2° and 19.5°. It is worth noting that a new diffraction peak appears at about 22° after adding CNCs to Zein/NPs, and the intensity of the diffraction peak increases with the increase in CNC content. The reason for this was that there was a strong characteristic peak at around 2θ = 22° of CNCs, with the increase in CNC content, this characteristic peak gradually appears in the XRD pattern of the films, which is also the evidence that CNCs have been successfully embedded into the film matrix. Huq et al. (2012) found a similar phenomenon when incorporating CNCs into alginate to prepare nanocomposite films [20]. Ye et al. (2017) also observed a new diffraction peak in the gelatin-trans-anethole/β-cyclodextrin inclusion complex film, which was attributed to the peak of the inclusion complex [35]. In addition, the incorporation of CNCs into Zein/NPs resulted in the presence of an additional diffraction peak and the peak intensity of the film at about 20° became stronger, relative to the contribution of CNCs that allowed an increase in the crystallinity of the films, which was beneficial to improving the barrier and mechanical properties of the nanocomposite films [20].

### 3.2. Physical Properties of Films

#### 3.2.1. Thickness, MC, WS and SD

As shown in Table 1, the thickness of films increased significantly after CNCs were added (*p* < 0.05), because of the increase in solid content in the film. Moreover, it can be seen from the table that with the increase in the content of CNCs, the MC and WS of the nanocomposite film decreased significantly (*p* < 0.05). The reason was that the filling effect of CNCs and the formation of hydrogen bond network increased the crystallinity of the film, resulting in the compact structure of the film and the reduction in free volume, so the equilibrium MC decreased [36]. The reason for the decrease in WS may be that CNCs were insoluble in water, and the decrease in WS was helpful to improve the water resistance of the nanocomposite film. The incorporation of CNCs significantly reduced the SD of the nanocomposite film (*p* < 0.05). Although there were hydroxyl groups in CNCs, which were hydrophilic, the hydrophilicity of hydroxyl groups was weakened by the three-dimensional network structure formed by hydrogen bonds. At the same time, the polymer chains were closely arranged, forming hydrogen bonds with water molecules, limiting the penetration and diffusion of water in film, thus preventing water from entering the film. Therefore, the addition of CNCs reduced the SD of the nanocomposite film.

#### 3.2.2. WVP

WVP is one of the most important performance indicators for controlling the transmission of water vapor through the film in food packaging [37]. As shown in Table 2, the WVP decreased with the increase in CNC content. The WVP of control Zein/NPs (without CNCs) was 3.27 × 10^−7^ g m^−1^ h^−1^ Pa^−1^. Compared with the control film, all the Zein/NPs films loaded with CNCs had lower WVP (i.e., 2.63, 2.2, 1.75 and 1.29 × 10^−7^ g m^−1^ h^−1^ Pa^−1^ for loadings of 2, 4, 6 and 8 wt%, respectively). Moreover, only 8% of CNCs were incorporated, and WVP was significantly reduced by more than 60%. Compared with pure zein film, the WVP of the nanocomposite film in this study was reduced by about 74% [15]. Sánchez-García, Hilliou and Lagarón (2010) also reported that the WVP was reduced to 71% compared to the control after the incorporation of CNCs into carrageenan [38]. The presence of CNCs was believed to increase the tortuosity of the nanocomposite film, resulting in a slower diffusion process, thereby reducing permeability. CNCs with negative charge have good dispersion in the matrix due to electrostatic repulsion, so the barrier properties were enhanced [20]. In addition, the results of FT-IR showed that the CNCs and the film matrix formed intermolecular hydrogen bonds, and the three-dimensional network structure formed by the hydrogen bonds hinders the diffusion of water vapor.

#### 3.2.3. Optical Property

**Figure 4:**
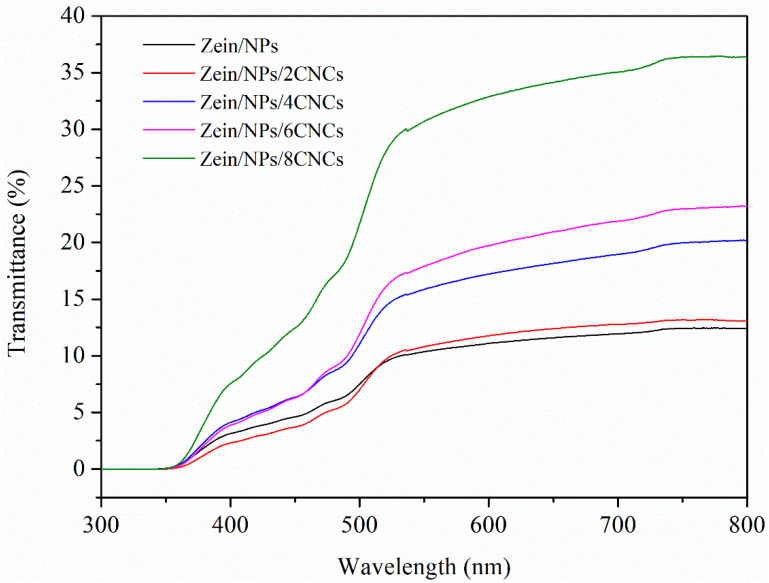
UV-vis light transmittance of Zein/NPs, Zein/NPs/2CNCs, Zein/NPs/4CNCs, Zein/NPs/6CNCs and Zein/NPs/8CNCs.

The light transmittance of the films was shown in Figure 4. It can be seen from the figure that the light transmittance of Zein/NPs was less than 10%. However, after adding CNCs, the transmittance of the nanocomposite film increases gradually, indicating that the protein network structure formed by the cross-linking of CNCs and zein was more uniform and orderly, which was more beneficial to improve light transmittance. This was consistent with the results of the morphology analysis of the films; after adding CNCs, the microstructure of the nanocomposite film became smooth and uniform. The transmittance of all kinds of zein films in UV region was close to zero, indicating that the films have a strong absorption ability to UV light. The main reason for this was that zein contains many aromatic amino acids, and its benzene ring structure has a strong ability to absorb UV light [39]. UV radiation protection is important not only for the protection of packaged foods, but also for the protection of packaging materials, as UV radiation can lead to the degradation of polymer materials [40]. Thus, nanocomposite films have the potential to be developed into UV protection materials.

The opacity of the films is presented in Table 2. The higher value of opacity indicates lower transparency, and vice versa. The opacity value of Zein/NPs was 5.954 ± 0.036, which was evidently higher than that of Zein/NPs with CNCs. With the increase in CNC content, the opacity of the Zein/NPs/CNCs film decreased significantly (*p <* 0.05), indicating that the transparency of the composite film increased. It is reported that improving the dispersion of nanocomposites within the matrix reduced the opacity of the film [41,42]. The esterified CNCs can be well dispersed in the matrix due to electrostatic repulsion, which was conducive to improving the transparency of the film.

#### 3.2.4. Mechanical Properties

As shown in Table 2, the TS of the Zein/NPs was 12.66 ± 0.33 MPa. The addition of CNCs resulted in a significant (*p* < 0.05) increase in TS. With 6 wt% CNCs, the TS of the film increased to 37.82 ± 1.07 MPa, which was 3 times of Zein/NPs. However, with 8 wt% CNCs, the TS of the film decreased slightly. Huq et al. (2012) also found that after the incorporation of 8 wt% CNCs in alginate-based film, the TS decreased slightly than film with 5 wt% CNCs [20]. The EAB of the Zein/NPs was 4.5 ± 0.16%. With the increase in CNCs content, the EAB of the films increased. With 2 wt% CNCs, the EAB of the film increased to 4.76 ± 0.11%, an increase of 5.8% compared to the Zein/NPs. Moreover, the incorporation of 4 and 6 wt% CNC contents raised the EAB of the nanocomposite films by 15.6% and 2%, respectively. However, with 8 wt% CNCs, the EAB of the nanocomposite film was even lower than Zein/NPs. An appropriate amount of CNCs (2–6 wt%) can improve the mechanical strength of the nanocomposite film. The mechanical properties of biodegradable films are generally related to the intermolecular forces and network structure [30]. The distribution and concentration of intra- and inter-molecular interactions have an important impact on the mechanical properties of protein-based films [15,30]. The esterified CNCs with negative charge can be evenly distributed in the film matrix due to electrostatic repulsion. The dense three-dimensional network structure formed by intermolecular hydrogen bonds between the CNCs and film matrix can improve the mechanical properties of the film. This phenomenon was evident from the SEM images. However, as the content of CNCs continues to increase to 8 wt%, the crystallinity of the nanocomposite film continued to increase, which reduces the flexibility of polymer molecules, resulting in the increase in brittleness and decrease in EAB [43]. Other researchers found a similar phenomenon when CNCs were incorporated into alginate film [20].

### 3.3. Antioxidant Properties

Antioxidant capacity, especially the ability to scavenge free radicals, is one of the basic characteristics of active packaging, because free radicals can cause oxidative damage and corruption of food [44]. The DPPH free radical scavenging activity and total antioxidant activity of the films after 3 days and 90 days of storage are shown in Table 3. The addition of CNCs had no significant effect on the DPPH free radical scavenging activity and total antioxidant activity of the films after 3 days of storage (*p* > 0.05). After 90 days, as the content of CNCs increased, the DPPH free radical scavenging activity of the films increased significantly (*p <* 0.05) and total antioxidant activity of the films increased slightly. The DPPH free radical scavenging activity of Zein/NPs/8CNCs was higher than that of Zein/NPs and still maintained about 68.94% after 90 days of storage, while the DPPH free radical scavenging activity of Zein/NPs only retained about 59.7%. In previous studies, only 22% of DPPH free radical scavenging activity and 44% of total antioxidant activity were retained in zein film containing CA after 90 days of storage [15], which was much lower than the antioxidant activity in this study. This difference in antioxidant activity was due to the fact that CA was effectively preserved against oxidization due to the formation of an inclusion complex between CA and β-CD [12]. Moreover, as the content of CNCs increases, relative to the contribution of CNCs that allowed increasing the crystallinity of the films, making the film structure more compact, which was conducive to blocking oxygen in the air and reducing the oxidation of active components. It should be noted that the DPPH radical scavenging activity and total antioxidant activity of the films stored for 90 days decreased compared with the films stored for 3 days. The CA in film was responsible for the antioxidant activity because polyphenol compound CA can improve scavenging activity and antioxidant activity [45]. Polyphenol compounds are composed of one or more aromatic rings bearing hydroxyl groups and are therefore potentially able to quench free radicals by forming resonance-stabilized phenoxyl radicals [46]. Because the film was exposed to air for a long time (90 days), oxidation resulted in the loss of CA in film to a certain extent, which decreased the antioxidant activity. Wang et al. (2013) also found that the total phenolic content in chitosan/tea polyphenols film decreased significantly after storage for 24 days, which was attributed to the oxidation of tea polyphenols in air; thus, the DPPH radical scavenging activity of the film decreased [47].

### 3.4. Oxidative Stability of Soybean Oil in Film Pouches

#### 3.4.1. PV

**Figure 5:**
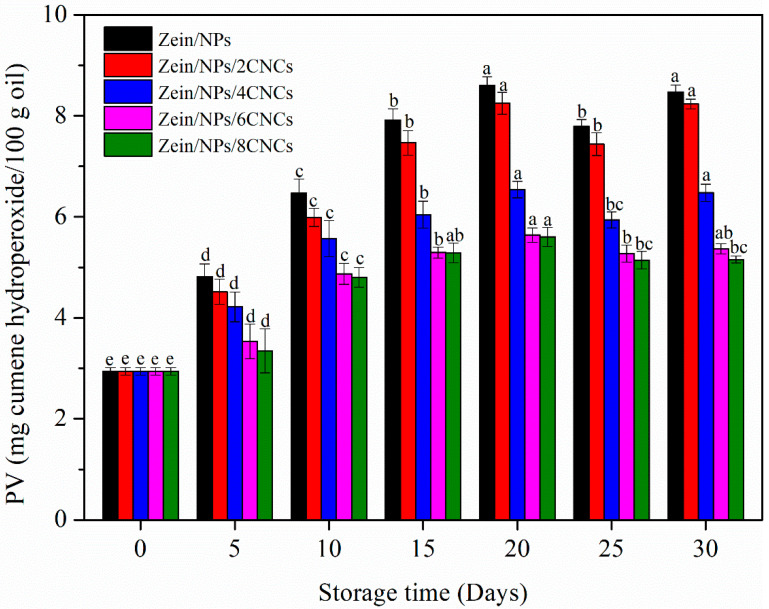
PV of oil packaged in Zein/NPs, Zein/NPs/2CNCs, Zein/NPs/4CNCs, Zein/NPs/6CNCs and Zein/NPs/8CNCs pouches. Different lowercase letters within the same packaging indicate significantly different as determined by Duncan’s test (*p* < 0.05).

For oil-containing products and edible oil, oxidative rancidity is the main problem [48]. After oxidative rancidity, the oil produces substances that are harmful to human health, such as peroxides, ketones and aldehydes [31]. PV is usually used to evaluate the primary oxidation of oil [49]. The PV of oil packaged in film pouches during storage is shown in Figure 5. In the first 10 days, the PV of all samples increased sharply, indicating that the oil was in the stage of lipid oxidation and reproduction. The reason is that lipid free radicals can react with oxygen to generate peroxyl free radicals, which become fast-reacting chain carriers by attacking new lipid molecules [31,49]. However, the PV of oil decreased after storage for 20 days, then increased slightly, and fluctuated among 20 and 30 days of storage. This was because hydroperoxides were decomposed into secondary oxidation products, which reduces the PV [49]. As compared with Zein/NPs, oil in films with CNCs displayed lower PV throughout 30 days of storage. Moreover, oil packaged in Zein/NPs/8CNCs displayed the lowest PV. This may be due to the improved crystallinity and denser structure of the films after the addition of CNCs, which can effectively prevent the oxygen in the air from entering the film to oxidize the oil. Nilsuwan et al. (2019) also found that chicken skin oil packaged in gelatin pouches had lower PV than that packaged in a low-density polyethylene pouch, which was related to the excellent oxygen barrier property of gelatin-based films [49]. Cho et al. (2010) also reported that enhanced oxygen barrier properties of zein films were beneficial in reducing the PV of olive oil after storage [50].

#### 3.4.2. TBARS

**Figure 6:**
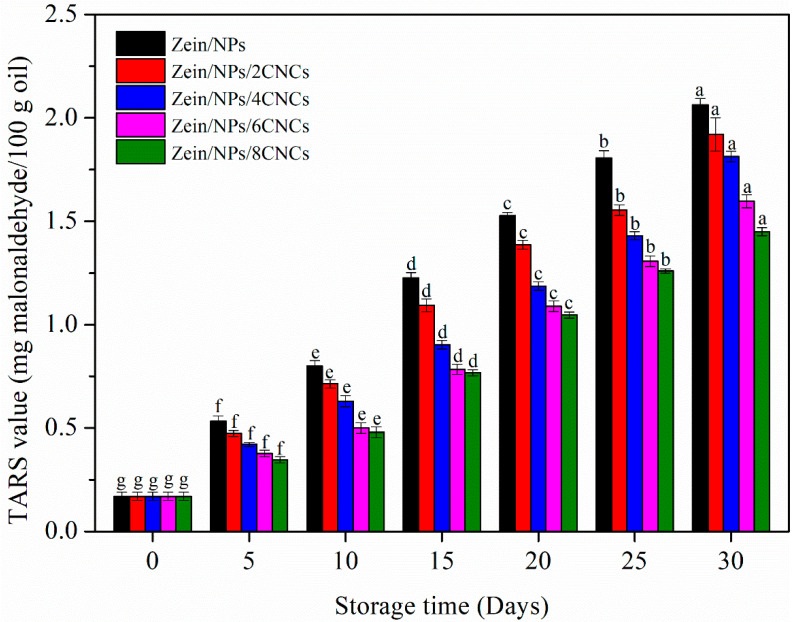
TBARS value of oil packaged in Zein/NPs, Zein/NPs/2CNCs, Zein/NPs/4CNCs, Zein/NPs/6CNCs and Zein/NPs/8CNCs pouches. Different lowercase letters within the same packaging indicate significantly different as determined by Duncan’s test (*p* < 0.05).

The secondary oxidation stage of oil can be studied by the determination of TBARS levels [51]. Figure 6 shows the changes of TBARS value of oil during storage. The TBARS value of all samples showed an upward trend during 30 days of storage, indicating that ketones and aldehydes were produced during the storage [31]. The increase in the TBARS value revealed the formation of secondary oxidation products, because TBARS value is an index of decomposition of hydroperoxides into the secondary oxidation products in later stages of lipid oxidation [52]. Hydroperoxides are decomposed to malonaldehyde, resulting in the off-flavor of oxidized lipids [53]. It is worth noting that the TBARS value of oil in Zein/NPs was higher than that in films with CNCs throughout 30 days of storage. The reason was that the films containing CNCs can more effectively inhibit the oil oxidation, thus reducing the amount of hydroperoxides decomposed into secondary oxidation products. Since Zein/NPs/CNCs films had better barrier properties than Zein/NPs, Zein/NPs/CNCs film packaging improved the oxidative stability of soybean oil. Nilsuwan et al. (2019) also found that the enhanced barrier properties of protein-based films could improve the oxidation stability of oil in packaging films [49].

## 4. Conclusions

In the present work, Zein/NPs active food packaging containing different amounts (0–8 wt%) of maleic-anhydride-modified CNCs were prepared by a facile solvent casting approach. SEM images showed that the addition of CNCs made the microstructure of the film more smooth and uniform, indicating that zein and CNCs have good compatibility. The FT-IR results showed that the CNCs could interact with film matrix through intermolecular hydrogen bonds. The results of XRD also evidenced the appearance of crystalline peak due to the existence of CNCs inside the films. The incorporation of CNCs in zein film can improve the barrier and mechanical properties. However, when the amount of CNCs was 8 wt%, the increase in crystallinity of the film leads to the increase in brittleness and the decrease in flexibility; thereby the EAB decreases significantly (*p* < 0.05). The addition of CNCs slightly improved the antioxidant stability of the nanocomposite films. Moreover, the oxidation stability of soybean oil was effectively improved by packaging in Zein/NPs with CNCs. Therefore, the results showed that Zein/NPs with CNCs can be used as active packaging materials to protect food from oxidation and prolong the shelf life of food products.

## Figures and Tables

**Table 1:** Thickness, MC, WS and SD of zein films.

Films	Thickness (mm)	MC (%)	WS (%)	SD (%)
Zein/NPs	0.132 ± 0.002 ^e^	21.82 ± 0.49 ^a^	14.46 ± 0.34 ^a^	15.7 ± 0.74 ^a^
Zein/NPs/2CNCs	0.139 ± 0.003 ^d^	18.62 ± 2.01 ^b^	12.39 ±0.22 ^b^	15.21 ± 1.53 ^a^
Zein/NPs/4CNCs	0.151 ± 0.001 ^c^	17 ± 1.29 ^cd^	12.19 ± 0.46 ^b^	13.5 ± 1 ^ab^
Zein/NPs/6CNCs	0.16 ± 0.002 ^b^	16.35 ± 0.11 ^d^	11.75 ± 0.78 ^b^	11.87 ± 1.24 ^b^
Zein/NPs/8CNCs	0.166 ± 0.002 ^a^	15.73 ± 0.26 ^e^	10.31 ± 0.17 ^c^	8.25 ± 1.62 ^c^

Different letters in the same column indicate significantly different as determined by Duncan’s test (*p* ˂ 0.05).

**Table 2:** WVP, TS and EAB of zein films.

Films	WVP (×10^−7^ g m^−1^ h^−1^ Pa^−1^)	TS (Mpa)	EAB (%)	Opacity
Zein/NPs	3.27 ± 0.07 ^a^	12.66 ± 0.33 ^d^	4.5 ± 0.16 ^c^	5.954 ± 0.036 ^a^
Zein/NPs/2CNCs	2.63 ± 0.05 ^b^	22.64 ± 1.77 ^c^	4.76 ± 0.11 ^b^	5.881 ± 0.029 ^b^
Zein/NPs/4CNCs	2.2 ± 0.12 ^c^	29.47 ± 1.95 ^b^	5.2 ± 0.1 ^a^	4.97 ± 0.031 ^c^
Zein/NPs/6CNCs	1.75 ± 0.11 ^cd^	37.82 ± 1.07 ^a^	4.6 ± 0.16 ^bc^	4.668 ± 0.043 ^d^
Zein/NPs/8CNCs	1.29 ± 0.11 ^d^	31.14 ± 1.24 ^b^	4.16 ± 0.11 ^d^	3.096 ± 0.035 ^e^

Different letters in the same column indicate significantly different as determined by Duncan’s test (*p* ˂ 0.05).

**Table 3:** DPPH radical scavenging activity and total antioxidant activity of films.

	DPPH Radical Scavenging Activity (%)		Total Antioxidant Activity (%)	
	Storage Time (Days)			
Films	3	90	3	90
Zein/NPs	87.41 ± 1.18 ^a^	59.7 ± 2.4 ^d^	91.5 ± 0.85 ^a^	80.04 ± 2.22 ^a^
Zein/NPs/2CNCs	87.6 ± 1.73 ^a^	61.39 ± 1.07 ^cd^	91.64 ± 1.37 ^a^	81.1 ± 2.02 ^a^
Zein/NPs/4CNCs	87.23 ± 1.28 ^a^	64.43 ± 1.34 ^bc^	92.19 ± 2.24 ^a^	81.9 ± 1.91 ^a^
Zein/NPs/6CNCs	87.9 ± 1.61 ^a^	67.47 ± 2.4 ^ab^	91.96 ± 2.13 ^a^	82.86 ± 2.56 ^a^
Zein/NPs/8CNCs	87.35 ± 2.72 ^a^	68.94 ± 2.33 ^a^	92.1 ± 1.99 ^a^	83.35 ± 3.83 ^a^

Different letters in the same column indicate significantly different as determined by Duncan’s test (*p* ˂ 0.05).

## Data Availability

Not applicable.
